# 1389. Parasitic Diseases Surveillance in Kazakhstan: Incidence Trends and Future Projections

**DOI:** 10.1093/ofid/ofad500.1226

**Published:** 2023-11-27

**Authors:** Ulyana Kirpicheva, Zhanna Shapiyeva

**Affiliations:** ​Scientific and Practical Centre for Sanitary-Epidemiological Expertise and Monitoring, Almaty, Almaty, Kazakhstan; SPC SEEM - the branch of NCPH, Almaty, Almaty, Kazakhstan

## Abstract

**Background:**

The COVID-19 pandemic has had a significant impact on healthcare systems worldwide, leading to a weakening of surveillance for parasitic diseases during quarantine measures in many countries. In 2022, this effect was observed in the Republic of Kazakhstan, where a significant increase in parasitic diseases was reported.

**Methods:**

The prediction of parasitic incidence was carried out based on official statistical data on the incidence of the population of Kazakhstan from 2011 to 2022. Exponential smoothing was used for incidence forecasting with 95% CI. Forecasts were carried out for echinococcosis, opisthorchiasis, ascariasis, enterobiasis, and giardiasis.

**Results:**

In 2022 there was a significant increase in parasitic diseases among the population of the Republic of Kazakhstan, with a total of 9,026 cases registered. Most cases were helminthiasis, accounting for 85% of all cases, while intestinal protozoal invasions accounted for 15%. The most prevalent parasitic diseases were enterobiasis, with an incidence rate of 24.9 per 100,000, followed by giardiasis with a rate of 6.8 per 100,000. Echinococcosis had an incidence rate of 3.9 per 100,000, while ascariasis and opisthorchiasis had rates of 6.4 and 2.5 per 100,000, respectively (Figure 1).

However, the expected incidence rates for parasitic diseases in 2023-2025 are projected to be lower than the long-term average incidence. There will be a significant decrease in the incidence rate of opisthorchiasis and enterobiasis. In 2023, the incidence rate of opisthorchiasis is expected to be 1.45 per 100,000, while the incidence rate of enterobiasis will be 12.1 per 100,000. This trend is projected to continue in 2024-2025 (Table 1).

**Figure:**
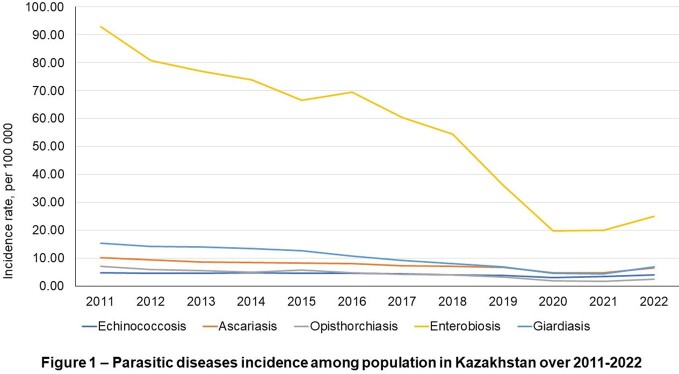

**Figure:**
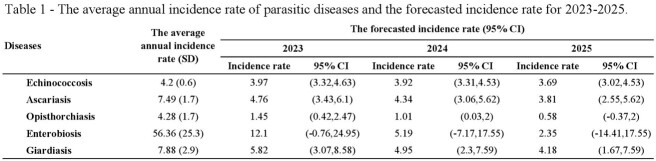

**Conclusion:**

As negative trends in parasitic diseases are observed, it is crucial to remain vigilant and take proactive measures to prevent the spread of these diseases. Following the results, additional measures were taken in the Republic of Kazakhstan to prevent echinococcosis and opisthorchiasis, such as strengthening the interdepartmental commission and enhancing epidemiological surveillance. It is essential to continue monitoring the situation and take proactive measures to prevent the spread of parasitic diseases.

**Disclosures:**

**All Authors**: No reported disclosures

